# Applied Endoscopic Anatomical Evaluation of the Lacrimal Sac

**Published:** 2015-05

**Authors:** Seyyed Mostafa Hashemi, Nezamoddin Berjis, Afrooz Eshaghian, Maryam Nejadnic, Akram Fereidani Samani

**Affiliations:** 1 *Department of Otorhinolaryngology, Isfahan University of Medical Sciences, Isfahan, Iran.*; 2 *Forensic Medical Center, Isfahan, Iran.*

**Keywords:** Anatomy, Dacryocystorhinostomy, Lacrimal Apparatus Diseases, Paranasal Sinuses, Surgery

## Abstract

**Introduction:**

Dacryocystorhinostomy (DCR), a popular surgical procedure, has been performed using an endoscopic approach over recent years. Excellent anatomical knowledge is required for this endoscopic surgical approach. This study was performed in order to better evaluate the anatomical features of the lacrimal apparatus from cadavers in the Isfahan forensic center as a sample of the Iranian population.

**Materials and Methods:**

DCR was performed using a standard method on 26 cadaver eyes from the forensic center of Isfahan. The lacrimal sac was exposed completely, then the anatomical features of the lacrimal sac and canaliculus were measured using a specified ruler.

**Results:**

A total of 26 male cadaveric eyes were used, of which four (16.7%) were probably non-Caucasian. Two (8%) of the eyes needed septoplasty, one (4%) needed uncinectomy, and none needed turbinoplasty. Four (16%) lacrimal sacs were anterior to axilla, one (4%) was posterior and 20 (80%) were at the level of the axilla of the middle turbinate. The mean difference of distance from the nasal sill to the anterior edge of the lacrimal sac (from its mid-height) was 39.04 (±4.92) mm. The mean difference of distance from the nasal sill to the posterior edge of the lacrimal sac (from its mid-height) was 45.50 (±4.47) mm. The mean of width and length of the lacrimal sac was 7.54 (±1.44) mm and 13.16 (±5.37) mm, respectively. The mean difference of distance from the anterior edge of the lacrimal sac to the posterior edge of the uncinate process was 14.06 (±3.00) mm, while the mean difference of distance from the anterior nasal spine to the anterior edge of the lacrimal sac (from its mid-height) was 37.20 (±5.37) mm.The mean height of the fundus was 3.26 (±1.09) mm. The mean difference of distance from the superior punctum to the fundus was 12.70 (±1.45) mm, and from the inferior punctum to the fundus was 11.10 (±2.02) mm.

**Conclusion:**

Given the differences between the various studies conducted in order to evaluate the position of the lacrimal sac, studies such as this can help to better identify the position of lacrimal sac during surgery based on ethnic differences. In addition, these studies can help novice surgeons to better navigate in a surgical scenario.

## Introduction

Dacryocystorhinostomy (DCR) is a popular surgical procedure first described using an endoscopic approach in 1989 (1). Advances in techniques, instruments and understanding of anatomy have improved surgical outcomes (2).

There are several indications for DCR, such as dacryocystitis, nasoorbitoethmoidal fractures, acquired epiphora, and congenital diseases.These disorders impair tear drainage, causing the tear to drain over the cheek (3). Balloon dilatation, external DCR and, more recently, endoscopic DCR, with or without silicone tubes, are performed to correct these obstructions (4–6). 

An anatomical evaluation is necessary for close surgical approach through the nose. Knowledge of the anatomy of the lacrimal apparatus within the nose is essential in the surgeon. A recognition of the relationship between the lacrimal sac, middle turbinate, and other anatomical landmarks such as the maxillary line, uncinate process and inferior turbinate has been shown to improve endoscopic techniques and outcomes (7). While these anatomic landmarks have great importance for the surgeon, if they become obscured by blood, they may be misidentified. 

There are a number of validated scales and measurements for the sinuses, but these may vary depending on the ethnic group. This study was performed in order to evaluate anatomical measurements of the lacrimal apparatus in the cadavers of the Isfahan forensic center as a sample of the Iranian population.

## Materials and Methods

DCR was performed using a standard method on 26 cadaver eyes in the forensic center of Isfahan. The study was approved by the Isfahan University Research Ethics Board and all procedures were performed in accordance with the 1964 Declaration of Helsinki. Cadavers were excluded if there was any disruption of the lateral nasal wall, the medial wall of the orbit, or the medial orbital rim or if there was damage to the medial canthus and/or lacrimal sac.

**Fig 1:**
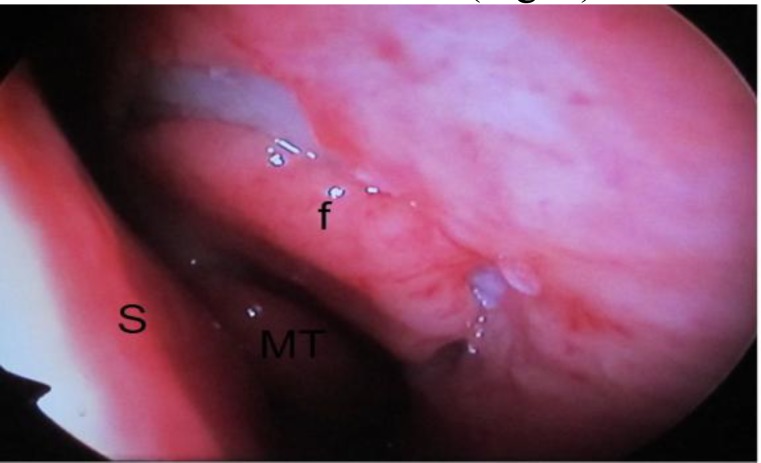
Left nasal cavity; elevation of lateral nasal mucosa anterior to the middle turbinate (f: elevate flap, MT: middle turbinate, S: septum)

Endoscopic DCR was performed by AE, who is in the last year of otolaryngology residency program.Under video endoscopic guidance, the nasal mucosa was incised approximately 5 mm superior and 10 mm anterior to the middle turbinate, using a crescent blade. Using a freer periosteal elevator, the nasal mucosa was elevated and the lacrimal bone removed (Fig. 1). 

**Fig 2:**
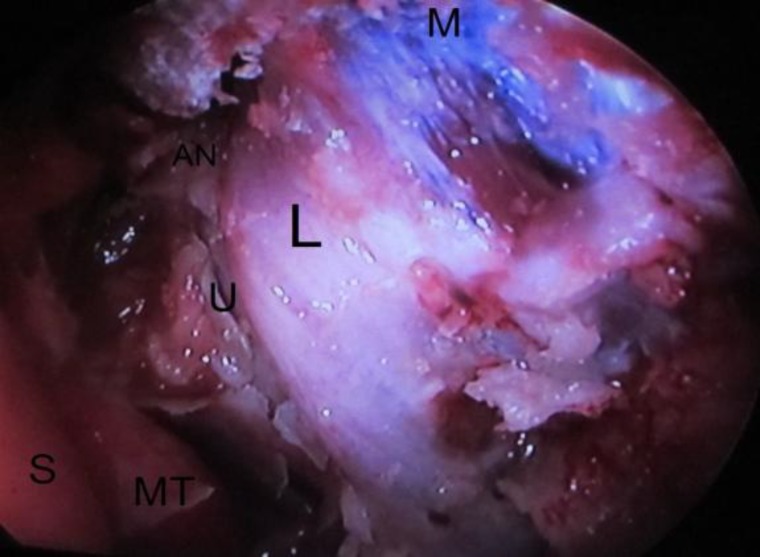
Left nasal cavity; exposure of lacrimal sac after lacrimal and maxillary bone removal (L: lacrimal sac, MT: middle turbinate, M: nasal muscles, AN: agger nasi, U: uncinate process, S: septum)

Next, an osteotomy was created using Kerrison rongeurs to expose the lacrimal sac completely. Then the inferior and superior punctum was dilated and probed (Fig.2).

**Fig 3:**
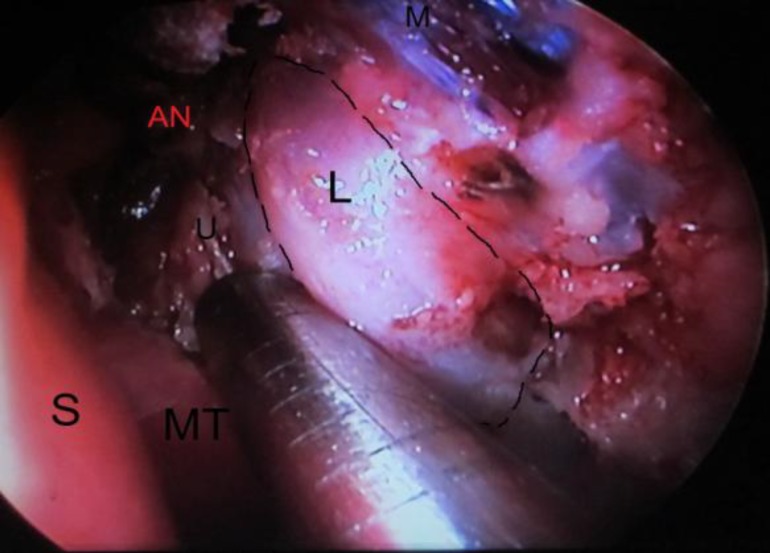
Left nasal cavity; exposure of lacrimal sac after lacrimal and maxillary bone removal (L: lacrimal sac, MT: middle turbinate, M: nasal muscles, AN: agger nasi, U: uncinate process, S: septum

Finally, the anatomical data of the lacrimal sac and canaliculus were measured using a specified ruler (Fig.3).

The distance from the nasal sill to the anterior edge of the lacrimal sac (from its mid-height), from the nasal sill to the posterior edge of the lacrimal sac (from its mid-height), from the nasal sill to the maxillary line (from its mid-height), from the nasal sill to the mid-point of the maxillary line (M- point), from the anterior edge of the lacrimal sac to the posterior edge of the uncinate process, from the anterior nasal spine to the anterior edge of the lacrimal sac (from its mid-height), from the superior punctum to the fundus, and the distance between the inferior punctum to the fundus, as well as the width of the lacrimal and height of the fundus sac, was measured. The precision of the ruler was 1 mm.

Data were analyzed using SPSS version 20 by descriptive methods. 

## Results

In total, 26 male cadaveric eyes were used, of which, four (16.7%) were more probably non-Caucasian. Two eyes (8%) needed septoplasty, one (4%) needed uncinectomy, and none needed turbinoplasty. Four (16%) lacrimal sacs were anterior to the axilla, one (4%) was posterior and 20 (80%) were at the level of the axilla of the middle turbinate. Thirteen were on the left side, and 11 were on the right side.

The distance between the nasal sill and the maxillary line (from its mid-height) was 36.06 (±4.44) mm (29–45 mm). The distance between the nasal sill and the mid-point of the maxillary line (M- point) was 37.33 (±5.15) mm (28–47 mm). 

There was a statistically significant difference between the right and left sides in fundus and sac height (P=0.01, P=0.045 respectively). No other significant differences in measurements between the right and left side were identified (Table. 1).

**Table 1:** Comparison of measurements between right and left side

		**Anterior nasal spine to anterior edge of lacrimal sac**	**Width**	**Anterior edge of lacrimal sac to agger nasi**	**Anterior edge of lacrimal sac to posterior edge of uncinate process**	**Inferior canaliculus**	**Superior canaliculus**	**Nasal sill to posterior edge of lacrimal sac**	**Nasal sill to anterior edge of lacrimal sac**	**Fundus height**	**Sac height**
Right	Mean (±SD)	36.00 (±5.00)	7.90 (±1.10)	11.60 (±3.33)	13.90 (±3.17)	10.50 (±2.03)	12.40 (±1.65)	45.30 (±4.66)	37.27 (±4.36)	3.87 (±1.24)	14.45 (±2.89)
	Minimum	25	6	7	9	7	10	37	28	2	10
	Maximum	41	10	16	18	13	15	51	42	6	19.5
left	Mean (±SD)	37.63 (±5.78)	6.91 (±1.44)	10.50 (±2.15)	13.70 (±2.97)	11.36 (±1.81)	13.04 (±1.35)	45.91 (±4.81)	40.41 (±5.48)	2.70 (±.54)	12.20 (±2.01)
	Minimum	28	5	7	8	8	11	39	33	1.5	9
	Maximum	48	10	15	18	14.5	15	57	50	3	16
P-value		0.486	0.093	0.362	0.877	0.307	0.335	0.765	0.145	0.010	0.045

## Discussion

A number of similar studies to this one are reported in the literature in other ethnic groups, but no reports on Iranian population have been published.

In a recent study in Canada, investigation of 10 cadavers using external or endoscopic osteomies of the lacrimal sac revealed similar dimensions irrespective of the technique used, but the endonasal opening was located slightly lower and more posterior the lateral nasal wall (8). This suggests that endonasal evaluation is more practical and necessary for endoscopic surgical guidance. 

In another study of 40 lacrimal pathways from 20 human cadavers in Brazil, transillumination of the common canaliculus revealed that the most common position of the lacrimal sac was between the free border of the middle turbinate and its insertion immediately underneath it. This is consistent with our results. The authors of the Brazilian study detected a maxillary line in 95% of the cases. In total, 12.5% of cases needed septoplasty, 35% needed unicifectomy, and 7.5% required middle turbinectomy (9). These findings are comparable to our study, in that two (8%) of our cases needed septoplasty, one (4%) needed uncinectomy, and none required turbinoplasty. 

In a study in 25 cadaveric specimens conducted in the USA, extranasal and intranasal measurements of the lacrimal crests, sac and duct, the suture line between the maxillary and lacrimal bones, and the maxillary sinus ostium were taken. The mid-point of the maxillary line (M-point) was used for reference. The maxillary line is defined as the junction of the uncinate and maxilla intranasally and the suture line is between the lacrimal bone and maxilla within the lacrimal fossa extranasally. This suture is approximately half-way between the anterior and posterior crests. The plane of the M-point corresponds to the superior margin of the maxillary sinus ostium posteriorly (average 10 mm) axially, and is just inferior to the lacrimal sac-duct junction anteriorly. The M-point is approximately 3.9 cm from the nasal sill in women and 4.8 cm in men in living patients (10). In our study, the distance between the nasal sill and the M- point was 37.33 (±5.15)mm(28–47 mm).

In a report of 36 Korean cadaveric eyes for investigation of the nasolacrimal duct under a surgical microscope, the location of the orifice of the nasolacrimal duct was at a mean of 17.5±3.1 mm from the limen nasi, 22.8±4.8 mm from the anterior nasal spine, and 21.4±3.5 mm from the axilla of the middle nasal concha (11). We reported these data for the lacrimal sac as an important component of endoscopic DCR.

 In the Korean population, the most frequent position of the lacrimal sac was posterior to the axilla of the middle nasal concha in 23 cases (64%). Our study revealed the most common place to be at the level of the axilla (80%). In the Korean population, this remarkable overlap of the nasolacrimal duct with the maxillary line was observed in 24 cases (67%) (11).

In another study of 50 specimens of formalin-fixed adult cadavers of both sexes of Indian origin, on the right side the mean length of lacrimal sac and nasolacrimal duct was 10.5 mm (1.04) and 16 mm (2.6) respectively; the mean breadth of the right lacrimal sac and nasolacrimal duct was 6 mm (0.63) and 5.66 mm (0.81) respectively. On the left side, the mean length of the lacrimal sac and nasolacrimal duct was 10.57 mm (1.13) and 16.42 mm (2.29), and the mean left breadth of the same structure was 6.71 mm (0.95) and 5 mm (0.81), respectively. The right and left sides did not show any significant statistical difference. There were no variations in the gross structure of the lacrimal sac or nasolacrimal duct (12). These results are partly similar to our study, except in terms of fundus and sac height.

## Conclusion

The findings of this study are different to some extent from those of other populations in previous reports. Given the differences in measures of the position of the lacrimal sac reported in different reports, studies such as this can help to better identify the position of the lacrimal sac during surgery, based on ethnic differences. In addition, these studies may help novice surgeons to better navigate surgical scenario.
